# Role of Hedgehog Signaling in Breast Cancer: Pathogenesis and Therapeutics

**DOI:** 10.3390/cells8040375

**Published:** 2019-04-25

**Authors:** Natalia A. Riobo-Del Galdo, Ángela Lara Montero, Eva V. Wertheimer

**Affiliations:** 1 School of Molecular and Cellular Biology, University of Leeds, Leeds LS2 9JT, UK; 2 Leeds Institute of Medical Research, School of Medicine, University of Leeds, Leeds LS2 9JT, UK; 3 Laboratorio de Transducción de señales y cáncer, Centro de Estudios Farmacológicos y Botánicos (CEFYBO-CONICET), Facultad de Medicina, Universidad de Buenos Aires, Buenos Aires C1121ABG, Argentina; ewertheimer@fmed.uba.ar

**Keywords:** hedgehog, breast cancer, clinical trial, sonic, Patched-1, Smoothened, glioma-associated oncogene, TNBC, therapeutics

## Abstract

Breast cancer (BC) is the leading cause of cancer-related mortality in women, only followed by lung cancer. Given the importance of BC in public health, it is essential to identify biomarkers to predict prognosis, predetermine drug resistance and provide treatment guidelines that include personalized targeted therapies. The Hedgehog (Hh) signaling pathway plays an essential role in embryonic development, tissue regeneration, and stem cell renewal. Several lines of evidence endorse the important role of canonical and non-canonical Hh signaling in BC. In this comprehensive review we discuss the role of Hh signaling in breast development and homeostasis and its contribution to tumorigenesis and progression of different subtypes of BC. We also examine the efficacy of agents targeting different components of the Hh pathway both in preclinical models and in clinical trials. The contribution of the Hh pathway in BC tumorigenesis and progression, its prognostic role, and its value as a therapeutic target vary according to the molecular, clinical, and histopathological characteristics of the BC patients. The evidence presented here highlights the relevance of the Hh signaling in BC, and suggest that this pathway is key for BC progression and metastasis.

## 1. Introduction

Breast cancer (BC), representing 1 in 4 cancer cases in women, has a global incidence of 2.1 million new cases per year (according to data from the World Health Organization) [1]. BC is the leading cause of cancer-related mortality, followed by lung cancer [1]. In addition to the high mortality rates, the care of patients with BC represents a heavy socioeconomic burden. A large proportion of women who undergo surgery, radiotherapy and/or chemotherapy suffer from their inability to work and care for their families. The global burden of the disease is estimated in disability-adjusted life years (DALYs), which combines the potential years of life lost due to premature death and the years of productive life lost due to disability. Recent figures from 2016 showed that BC caused 15.1 million DALYs during that year alone [2]. Given the importance of BC in public health, it is essential to identify biomarkers to predict prognosis, predetermine drug resistance and provide treatment guidelines that include personalized targeted therapies.

While the mortality rate of BC patients has steadily declined over the years owing to earlier detection and better targeted therapies, triple negative BC tumors (TNBC, tumors which lack hormone receptors and HER2 amplification) still have a poor prognosis due to their higher tendency to relapse [3]. The development and progression of metastatic disease is the main cause of death related to BC; therefore, increasing our mechanistic knowledge of BC metastasis is of fundamental importance for the development of future therapies targeting this stage of the disease.

The role of Hedgehog (Hh) signaling in normal breast tissue homeostasis and in BC pathophysiology is underscored by a growing number of publications [4,5,6]. The contribution of the Hh pathway in BC tumorigenesis and progression, its prognostic role, and its value as a therapeutic target vary according to the molecular and histological subtype of BC. In particular, Hh signaling appears to be a fundamental mechanism in TNBC [6,7,8,9,10]

In this comprehensive review we will discuss the role of Hh signaling in breast development and homeostasis and its contribution to tumorigenesis and progression of different subtypes of BC. We will also examine the efficacy of agents targeting different components of the Hh pathway both in preclinical models and in clinical trials.

## 2. Overview of Breast Cancer

BC is a heterogeneous disease at the histopathological, clinical and molecular level [11]. The heterogeneity at the molecular level has been demonstrated by reproducible differences in the frequencies of genomic aberrations and variations of the gene expression patterns among mammary carcinomas, even between tumors of the same histological type. The molecular profile of tumors is generally estimated from immunohistochemical studies that include the analysis of the expression of estrogen (ER) and progesterone (PR) hormone receptors, HER2 (ErbB2) tyrosine kinase receptors, and the nuclear marker of cell proliferation Ki-67. Whole genome expression studies using microarrays have led to classification of BC into five different subtypes of breast carcinomas based solely on clustered gene expression: luminal A (ER^+^ and/or PR^+^, HER2^−^, low Ki-67), luminal B (ER^+^ and/or PR^+^, HER2^−/+^, high Ki-67), HER2-overexpressing (ER^−^, PR^−^ and HER2^+^), basal-like (express markers of basal/myoepithelial cells), and normal breast-like (enriched in markers of adipose cells/normal mammary cells) [12]. Following the initial identification of five BC molecular subtypes, the advances in gene expression studies allowed the identification of different molecular entities, resulting in further subclassifications [13]. These subtypes reflect basic differences in tumor biology, in clinical parameters and in the survival of patients [14], allowing the development of prognostic patterns [15,16]. Currently, genomic platforms are used in the clinic to obtain the gene expression profiles of the tumors, which allow predicting the clinical evolution of the patient. The more broadly used platforms are Mammaprint, Oncotype DX, Mammostrat, Breast Cancer Index, EndoPredict, IHC4 and PAM50.

Although tumors of the same subtypes are more homogeneous and share similar clinical behaviors, they still display differential responses to therapeutic agents. These observations suggest that different types of BC originate from dysregulation of different oncogenes, paving the way for the search for highly specific therapeutic targets.

After diagnosis of BC, the choice of treatment depends on the disease stage at diagnosis and the molecular profile of each individual tumor [17]. The treatment plan may include chemotherapy, hormone therapy, targeted therapies, radiotherapy, and lumpectomy or mastectomy. For early-stage BC, the recommended course of treatment is surgery followed by radiation with or without systemic adjuvant therapy to prevent and reduce the growth of any undetected metastasis [18,19]. The choice of a specific treatment depends on the type of BC: ER and PR-positive tumors are treated with hormone-regulatory compounds, such as: (1) anti-estrogens (among them, selective modulators of ERs (SERM) or downregulators of ERs (SERD)); (2) analogues of the luteinizing hormone-releasing hormone (LHRH); (3) aromatase inhibitors; (4) estrogens and androgens. An alternative treatment is ovarian ablation, which involves removing or interrupting the function ovaries, the main source of estrogen. Tumors with positive hormone receptor status, characterized by upregulation of cyclin D1 and concurrent activation of cyclin-dependent kinases CDK4 and CDK6 [20], can also be treated with CDK4/CDK6 inhibitors palbociclib, ribociclib and abemaciclib. HER2-overexpressing tumors are generally treated with the monoclonal antibodies trastuzumab and pertuzumab. Lapatinib, a small molecule reversible competitive inhibitor of the tyrosine kinase activity of HER2/ErbB2 and EGFR [21,22], is also used to block HER2 signaling in this subtype. Everolimus, an analogue of rapamycin (rapalog) that inhibits signaling downstream of mTOR, is approved for treatment of ER-positive metastatic BC following the appearance of resistance to antiestrogen therapy [22].

The most challenging BC subtype is the triple negative breast cancer (TNBC), representing between 10 to 24% of cases [23]. TNBC is very heterogeneous and highly aggressive. It is noteworthy to mention that the term TNBC refers to the immunohistochemical description of the tumor (ER^−^, PR^−^, HER2^−^); while basal-like BC is defined from its gene expression signature and refers to a subset of TNBCs that express other markers of basal/myoepithelial cells such as basal cytokeratins (CK5/6, CK14 and CK17), vimentin, and epidermal growth factor receptor (EGFR). Hence, the terms are not synonyms and cannot be used interchangeably despite that 70–80% of TNBCs are also basal-like [24]. The nomenclature used hereafter will be based on the classification used in the referenced papers. In addition basal-like 1 (BL1) and basal-like 2 (BL2) subtypes, TNBCs can be further divided into immunomodulatory (IM), mesenchymal-like (M), mesenchymal stem-like (MSL), and luminal androgen receptors (LAR) subtypes based on gene expression signatures [24]. Because of the limited targeted therapeutic options and due to the absence of ER, PR, and HER2 and EGFR expression [24,25,26,27,28,29], TNBC is associated with poor prognosis. For this BC subtype, radiation, surgery, and cytotoxic chemotherapy with taxanes and anthracyclines are the main line of treatment. However, there is a high risk of relapse (40.7% of patients), and metastatic spreading [30] mainly to the brain and lungs [31]. Cisplatin and carboplatin have shown efficacy in extending overall survival and progression-free survival of TNBC patients with lung metastasis [32]. However, BL1 and BL2 usually develop resistance against chemotherapeutic agents [33].

Many promising clinical trials for the treatment of BC that include the use of progestogens, vaccines, checkpoint inhibitors/immune modulators, adoptive cell therapy (T cell transfer), therapies with oncolytic virus and antibodies are under development. Despite the available alternatives for BC treatment, metastatic disease is still of great concern. Therefore, identification of novel therapeutic targets and new agents that can modulate resistance to chemotherapy in BC is crucial.

## 3. The Hedgehog (Hh) Signaling Pathway

The Hedgehog (Hh) signaling pathway plays an essential role in embryonic development, tissue regeneration, and stem cell renewal [34,35,36,37]. The Hh protein family, consisting of Sonic (SHH), Indian (IHH) and Desert (DHH) Hedgehog, activates a signaling cascade by binding to the twelve-transmembrane (TM) receptors Patched1 and/or Patched2 (PTCH1 and PTCH2) leading to derepression of the seven-TM protein Smoothened (SMO). Derepression of SMO subsequently activates the glioma-associated oncogene (GLI) transcription factors (GLI1, GLI2 and GLI3), which in turn stimulate expression of downstream target genes [38,39]. GLI2 and GLI3 are constitutively expressed and serve as transcriptional activators (GLI-A) in their full-length form and as transcriptional repressors (GLI-R) after partial proteasomal processing [40,41]. In contrast, GLI1 is induced by GLI2 and GLI3 and acts exclusively as a strong transcriptional activator [42].

The activation of GLI-dependent transcription by Hh ligands constitutes the so-called “canonical” Hh signaling and requires an intact primary cilium. The primary cilium is a flagellar-like organelle that forms during interphase upon microtubule polymerization from the basal body and is resorbed during S phase (reviewed in reference [43]). Binding of a Hh ligand to PTCH1 in the primary cilium results in its inhibition and internalization, followed by accumulation of SMO in the ciliary membrane and activation of GLI2 and GLI3.

The cross-talk between Hh and other oncogenic pathways can also result in the activation of GLI1 and GLI2. Activation can be the consequence of posttranslational modifications [44] or transcriptional upregulation of the GLI proteins by Hh ligand-independent mechanism, sometimes referred to as a type of “non-canonical” Hh signaling [45]. However, non-canonical Hh signaling most commonly refers to Hh-dependent cellular responses that are independent of the GLI transcription factors, and in some cases, are also independent of the primary cilium [46]. Non-canonical Hh signaling can be divided into type I, in which Hh regulates PTCH1-specific functions that are independent of SMO (apoptosis, autophagy, proliferation), and type II, which depends on the function of SMO as a G-inhibitory protein-coupled receptor [47].

The canonical Hh pathway has been implicated in tumorigenesis and progression of many cancer types. Ligand-independent constitutive activation of GLIs (as a consequence of loss-of-function mutations of *PTCH1* or gain-of-function mutations of *SMO*) drives basal cell carcinoma (BCC) and Shh-type medulloblastoma development [48,49,50]. Even though epithelial cancers do not present mutations in the components of the Hh pathway, they are characterized by upregulation of SHH and/or IHH, which can modulate tumorigenesis in an autocrine [51,52], paracrine [53,54], or reserve-paracrine fashion [55]. This refers to Hh ligands produced by the cancer cells and acting cell-autonomously or on the stroma, or Hh proteins produced by the stroma acting on the cancer cells. Regardless of the mechanism of GLI activation, a high level of GLI-target gene expression has shown to contribute to tumorigenesis [56,57,58].

## 4. Involvement of Hh Signaling in BC: Molecular Mechanisms

### 4.1. Hh Signaling During Normal Mammary Gland Development

The mammary gland forms during embryonic development, grows during puberty and differentiates during lactation [59]. The components of the Hh pathway are differentially expressed in mammary tissue at different developmental stages [60,61,62,63,64,65]. During mammary gland morphogenesis, the canonical Hh pathway is repressed. Formation of the early mammary buds in mice requires the active repression of *gli1* by GLI3R. This was demonstrated by loss of mammary buds after forced expression of GLI1 in the mammary gland parenchyma and in mice deficient in GLI3 (*gli3^xt^*) [65]. Accordingly, embryos lacking SHH and IHH display normal development of mammary buds when transplanted into fat pads of wild type females [60,63].

Ductal morphogenesis during puberty appears to require specific levels of canonical and non-canonical Hh signaling. Ductal morphogenesis is impaired in mice carrying a mutation in the C-terminal domain of PTCH1 (*ptc1*^mes^) that affects type I non-canonical Hh signaling but not GLI activation [66]. However, transgenic mice that express constitutively active SMO, leading to very high canonical signaling activity, including upregulation of *Ptch2, Gli1, Gli2, Jag2* and *Dll-1*, and downregulation of *Notch4* and *Hes6*, develop mammary ductal dysplasia [67,68]. Type I non-canonical Hh signaling activates proliferative c-Src, ERα and ERK cascades in mammary luminal epithelial cells, leading to elongation of the terminal buds during puberty [66,69,70]. Primary cilia, present in luminal epithelial, myoepithelial and stromal cells, are necessary during early branching morphogenesis but later disappear from luminal epithelial cells in the mature mammary gland [71]. After the high activity during puberty, the Hh ligands, GLI1, GLI2, GLI3, and PTCH1 are expressed at low levels in the mature mammary gland [60,64,71].

These data indicate that the canonical Hh signaling pathway is mainly repressed during mammary embryonic development, that type I non-canonical Hh signaling plays an important role in ductal morphogenesis at puberty, and that the pathway is downregulated in the normal adult mammary tissue.

### 4.2. Canonical and Non-Canonical Hh Signaling in BC

Mutations in *SHH*, *PTCH1*, and *GLI1* are very rare in BC [5,72,73,74], arguing against mutational activation of the Hh pathway in BC. Multiple cancers have been associated with ligand-dependent activation of Hh signaling [75,76] by upregulation of SHH or IHH [77,78]. This seems to be the case in BC, in which aberrant upregulation of SHH has been reported in association with progression and changes in the tumor microenviroment [79]. On the other hand, and despite the published evidence of a role of type I non-canonical Hh signaling in mammary gland development [80], its contribution to BC tumorigenesis has not been investigated. Similarly, there is a lack of information on the potential role of type II non-canonical Hh signaling in BC, although its known functions in angiogenesis, cell migration and activation of small Rho GTPases [81,82,83] suggest that type II signaling could play an important role in the tumor stroma.

Despite the lack of mutations in Hh genes in BC, activation of the canonical Hh pathway in animal models results in BC. In one study, hyperactivation of the pathway by overexpression of GLI1 under the MMTV promoter in the mammary epithelium was sufficient to induce hyperplastic lesions and tumor development in mice [84,85]. Xenograft transplantation experiments revealed that SHH overexpression is associated with larger aggressive tumors, increased lymphatic invasion, and metastasis [79]. Moreover, SHH overexpression upregulated the pro-angiogenic transcription factor CYR61 in a GLI-dependent manner, contributing to the development of highly vascularized tumors [86].

### 4.3. Regulation of SHH in BC Cells

Since SHH expression regulates ligand-dependent Hh pathway activation in BC, obvious questions are how and why expression of SHH is upregulated. While several mechanisms might account for this, the *SHH* gene is known to be exquisitely regulated both temporally and spatially during embryonic development by genetic and epigenetic mechanisms. A candidate regulator of SHH expression in BC is the nuclear factor-kappa B (NF-κB) transcription factor [87,88]. NF-κB is an inflammatory signaling mediator that promotes cell proliferation, migration, differentiation and self-renewal in cancer [89,90]. NF-κB positively regulates SHH expression in a variety of cancer types, including BC [88,91,92,93]. It has been postulated that an NF-κB-binding element present within a normally methylated CpG island in the *SHH* promoter is accessible to NF-κB binding following demethylation. Reduced CpG methylation of the *SHH* promoter has been linked to increased SHH expression in several cancers [88,94]. Indeed, treatment of BC cell lines with 5-azacytidine, a DNA methylase inhibitor, diminished methylation of the *SHH* promoter and increased its expression [88,95]. Moreover, 5-azacytidine potentiated SHH upregulation following TNFα stimulation of BC cells (which activates NF-κB) but not when the NF-κB inhibitor PDTC was present [95]. These results suggest a concerted regulation of SHH expression with NF-κB in BC at both transcriptional and epigenetic levels.

### 4.4. PTCH1 Expression in BC Cells

While PTCH1 is a receptor and acts as a negative regulator of Hh signaling, its expression is upregulated by GLI-dependent transcription and thus it serves as a surrogate marker of canonical Hh signaling activation [47]. The normal low expression level of PTCH1 and the lack of commercial antibodies with enough sensitivity to detect endogenous protein prevent an accurate quantification of its level in BC tumors by immunostaining. However, PTCH1 expression at the mRNA level was found to be reduced in the MCF7 BC cell line in correlation with promoter hypermethylation [96]. In disagreement, another study reported increased PTCH1 expression in the same cell line and also in T47D, 13762 MAT B III, and SKBR3 cells using radiolabeled SHH protein binding [97]. However, SHH can bind with high affinity to a number of receptors other than PTCH1, such as PTCH2, HHIP, GAS1, CDON, and BOC [47], which complicates the interpretation of those findings. To be able to elucidate PTCH involvement in BC and its therapeutic potential, further studies should address the discrepancies among authors and clearly determine if BC features can be associated or not to a dysregulation of PTCH1 expression.

### 4.5. GLI1 Expression in BC

As will be discussed later in this review, overwhelming evidence supports the involvement of GLI1 and GLI2 target genes in BC proliferation, survival, migration, invasion, EMT, angiogenesis, and osteolytic metastasis [7,8,79,86,98,99,100,101]. Conversely, there are no reports supporting an oncogenic role for GLI3 in BC.

Independent studies reported a higher expression of GLI1 in TNBC and basal-like BC cell lines and TNBC tumors compared to other BC subtypes and to normal mammary epithelial cells and breast tissue [10,100,102,103]. Silencing of GLI1 expression with *si*RNA or inhibition of SMO with the plant-derived alkaloid cyclopamine in two ER-negative, one TNBC and one basal-like BC cell line reduced proliferation and invasion [102]. Similar experiments in SUM145 TNBC cells showed increased apoptosis and reduced migration after GLI1 silencing [100]. In contrast, the ER-positive cell lines T47D and MCF7 show a lower GLI1 expression level than observed in TNBC [10].

#### Truncated GLI1 in BC

A GLI1 mRNA splice variant that eliminates 41 codons spanning exon 3 and part of exon 4 results in expression of a truncated form of GLI1 (tGLI1) [104]. tGLI1 was detected both in BC cell lines and primary tumors, but not in normal breast tissue. This shorter variant of GLI1 does not display increased transcriptional activity against naked GLI-binding sites, as determined by transfection of an 8XGli-BS-luciferase reporter into BC cells [104]. However, tGLI1 is able to enhance the expression of selective GLI target genes, such as VEGF-A, CD24, MMP-2 and MMP-9 in BC cells. In agreement, BC cells expressing tGLI1 migrate faster, are more invasive and more pro-angiogenic than cells expressing the longer GLI1 variant. tGLI1 also upturn anchorage-independent growth of BC cells [104]. These results suggest that expression of tGLI1 might increase the metastatic potential of BC cells.

### 4.6. LKB1 as a Negative Regulator of Hh Signaling in BC

Liver kinase B1 (LKB1) is a tumor suppressor that regulates autophagy and cell growth through direct phosphorylation and activation of the AMP-dependent protein kinase AMPK [105]. LKB1 has been shown to inhibit Hh signaling and impair xenograft growth of MDA-MB-231 BC cells, while knockdown of LKB1 increased both Hh signaling and the rate of xenograft growth [106]. In agreement with the in vitro and animal studies findings, biopsies of ductal carcinomas showed an inverse correlation of the expression level of LKB1 with SHH, GLI1 and SMO [106]. Thus, available evidence suggests that LKB1 acts as a negative regulator of the canonical Hh pathway in BC.

### 4.7. Estrogen Receptor-Positive BC and Hh

Estrogen (17β-estradiol) is a fundamental regulator of ER-positive BC development and progression. Estrogen increases cell proliferation and particularly boosts the proportion of cancer stem cells (CSC) within a cancer cell population [99,107,108,109]. The CSC compartment is proposed to promote metastatic spreading of the tumor and resistance to anti-hormone therapy. A recent study showed that estrogen increases the levels of GLI1 and GLI2 in several ER-positive BC cell lines [110], whereas inhibition of GLI1 and GLI2 activity with GANT61 reduced the number of mammosphere-forming CSC in the cultures. These results support a role of GLI transcription factors as mediators of the effect of estrogen in BC [110]. Another study showed that estrogen increases SHH and GLI1 expression leading to activation of Hh signaling (determined by GLI1 nuclear translocation) and promotes invasiveness in the ER-positive T47D (HER2-) and BT-474 (HER2+) cells [111]. These results suggest a crosstalk of ER and the Hh signaling pathways to increase invasiveness of ER-positive BC cells.

### 4.8. Hh Signaling in TNBC

There is a pressing need for novel therapies that target the highly aggressive TNBC, which is defined by absence of ER, PR and HER2 immunostaining [24,112,113,114]. Due to its propensity to metastasize [115,116] and to its intrinsic chemoresistance [107,117], TNBC is thought to be highly enriched in CSCs [118,119,120,121]. The enrichment of the tumor with cells that harbor CSC markers (a CD44^+^/CD24^−/low^ and Claudin-low phenotype [122,123] and high aldehyde dehydrogenase 1 (ALDH1) activity [118]), and their ability for anchorage-independent growth and mammosphere formation [124,125]) support the notion that TNBCs are poorly differentiated and stem-cell like. Indeed, it has been shown that the CSC phenotype and genetic signature is associated with a higher incidence of relapse and metastasis [126] along with a poor pathological response to treatment [127].

Many lines of evidence support a role for canonical Hh signaling in TNBC. First, TNBC has a high proportion of basal-like progenitors, which were shown to retain primary cilia, and express GLI1, suggesting that TNBC might be responsive to ligand-dependent stimulation of canonical Hh pathways [128,129]. Second, overexpression of SHH in TNBC increases cell proliferation, colony formation, migration and invasion in vitro [86,130]; promotes orthotopic xenograft growth in the mammary pad, and increases spontaneous lung metastasis in vivo [86]. However, Hh-independent upregulation of GLI1 by NF-κB and activation of GLI2 by FOXC1 have been reported in TNBC [7,131], suggesting that there is more than one pathway leading to GLI activation in TNBC. Activation of GLI1 might be involved in increased cancer cell resistance to doxorubicin, paclitaxel, and cisplatin by upregulation of the multidrug resistance protein-1 (MDR-1) [132].

Canonical Hh signaling also contributes to TNBC growth and metastatic spread by enhancing tumor angiogenesis by several mechanisms, i.e., the increased expression of metalloproteases [86,104], CYR61 [86], and VEGF receptor 2 (VEGFR2) [129]. The central role of canonical Hh signaling in TNBC angiogenesis is also highlighted by the reduction of xenograft proliferation and vascularization caused by administration of NVP-LDE225, an FDA-approved SMO inhibitor [129]. In addition, a role of non-canonical Hh signaling in TNBC angiogenesis is possible, since it was demonstrated to promote endothelial cell tubulogenesis and survival [81].

The characteristic tendency of TNBC to form osteolytic bone metastasis might also be linked to the Hh pathway. In vitro experiments and transplant models in mice indicate a correlation between local tumor invasion [79], visceral [86] and osteolytic metastasis [101,133] and constitutive SHH expression in TNBC. In the bone niche, metastatic BC cells are exposed to TGF-β which upregulates GLI2 and, consequently, parathyroid hormone-releasing protein (PTH-rP), which plays a key role in osteolytic metastasis [101,133]. TGF-*β* upregulates GLI2 expression through binding of SMAD-3 and β-catenin to the *GLI2* promoter [134]. Metastatic BC cells also express RUNX2, a bone transcription factor that upregulates IHH and, in concert with SMAD activation by TGF-*β*, promotes osteolytic metastasis of the TNBC cell line MDA-MB-231 [135]. In addition, Hh proteins secreted by TNBC cells activate stromal osteoclast precursors in a paracrine fashion through pro-inflammatory cytokines, osteopontin (OPN), and matrix metalloproteases, leading to bone resorption [136,137]. Several studies in mice support the paracrine effect of Hh secreted by TNBC cells on stromal components that contributes to tumor growth, invasion and metastasis [79].

### 4.9. Hh Signaling in BC Stem Cells

The successful eradication of BC depends on the elimination of cancer stem cells (CSCs), which have the capacity to self-renew and regenerate the tumor [109]. Breast CSCs are characterized by expression of stemness markers like ALDH1, NOTCH, and OCT4 [138]. GLI1 not only is a stemness marker in many tissues [139], but it also has the capacity to upregulate SRY-box 2 (SOX2) and OCT4 expression in CSCs [140]. GLI1 is upregulated in breast CSCs by NF-κB and FOXC1, independently of Hh ligands [7,131]. In support, inhibition of NF-κB reduces GLI1 expression in several BC cell lines (BT549, HS578T, MDA-MB-231, MDA-MB-157, MDA-MB-436 and MCF10A) [7].

Not only CSCs have the capacity to regenerate a clonal tumor with progenitors and terminally differentiated cells [141,142,143], they are also more resistant to chemotherapy. Paradoxically, doxorubicin and cisplatin can lead to activation of the Hh pathway in breast CSCs, which increases MDR-1 expression and resistance [132,144]. The recent development of suspension cultures of mammary cells that form mammospheres enriched in CSCs, progenitors capable of self-renewal and terminal differentiation [145], and their transplantation into the cleared mammary fatpads of syngenic mice [146,147], has greatly advanced our understanding of breast CSC biology.

A comparison of mammosphere forming cells vs. terminally differentiated mammary cells isolated from normal breast tissue revealed that PTCH1, GLI1, and GLI2 are highly expressed in the CSC compartment, and that their expression is downregulated upon differentiation [148]. Stimulation of Hh signaling increased and inhibition with cyclopamine reduced the number of mammosphere-forming cells (i.e., CSCs), their proliferation rate [148], and their decreased ALDH1 activity [7,131]. The effect of SHH on the CSCs proliferation was shown to depend on activation of the GLI-dependent canonical pathway and to be partly mediated by the polycomb protein BMI1.

Altogether, these studies indicate that the upregulation of GLI1 is key for breast CSC maintenance and proliferation.

### 4.10. Hh Signaling in Epithelial-Mesenchymal Transition and Metastasis in BC

Primary tumor cells lose their epithelial polarized phenotype and acquire mesenchymal-like features during the process of invasion and metastasis, a phenomenon known as epithelial-mesenchymal transition (EMT) [149,150]. EMT is a complex transdifferentiation process regulated by upregulation of a group of transcription factors, such as Zeb1/2, Snail1, Snail2 (Slug), Twist 1/2, Goosecoid, FOXM1, FOXC1, and FOXC2, among others. It results in loss of the epithelial cell adhesion molecules E-Cadherin and Claudin and gain of expression of Vimentin and N-Cadherin, characteristic of fibroblasts [151,152]. EMT enhances not only invasiveness, but also self-renewal capacity and resistance to apoptotic stimuli [127,153,154,155,156]. Numerous studies support a positive role of Hh signaling in EMT in breast and other cancers [7,132,157,158,159,160,161]. Silencing of GLI1 in TNBC cells abrogates hypoxia-induced upregulation of Vimentin and loss of E-Cadherin [161]. Another report showed that GLI1 represses E-Cadherin expression in basal type B SUM145 cells but not through reduction of Snail, a classical E-Cadherin repressor [100]. In vivo animal studies also support a role of GLI1 in the EMT process in BC. Mammary ductal hyperplasia in transgenic mice expressing a constitutively active SMO mutant (SMO-M2) exhibits signs of loss of basolateral polarity reminiscent of EMT [68,162]. Mice expressing a conditional GLI1 transgene develop basal-like ER-negative tumors characterized by loss of E-Cadherin. In summary, the published evidence supports the notion that canonical and non-canonical activation of GLI1 promotes EMT.

Histopathological studies of ductal carcinomas in situ (DCIS) and invasive ductal carcinomas (IDC) support a role of GLI1 expression in acquisition of the invasive phenotype, since IDC samples show an increased % of cells with nuclear GLI1 staining [111]. The role of GLI1 in EMT in BC is further supported by its association with poor prognosis [103,163].

#### 4.10.1. EMT Contribution to Metastasis via the Hh Pathway

In recent years, the role of EMT in the metastatic process has been challenged by lineage-tracing studies that observed a lack of cells that had undergone EMT in the distal metastatic sites [164,165]. However, other researchers proposed a model in which intratumor heterogeneity results in cooperation of EMT-transformed cells to enable non-EMT cells to escape the primary site and reach secondary sites [166]. In support of this theory, weakly metastatic BC cells show enhanced metastatic potential by a paracrine effect of EMT cells. EMT cells (positive for Twist1, Snail1, and Six1) induce metastasis of Six1-negative cells by paracrine activation of GLI1-dependent transcription [166]. Indeed, pharmacological inhibition of GLI1 with GANT61, but not of SMO with IPI926, in non-EMT cells prevents the phenotypic changes caused by EMT-cells, suggesting that the paracrine effect is not mediated by Hh ligands [166].

While several SMO inhibitors are in clinical trials for the treatment of BC and other cancers, these findings suggest SMO inhibitors might fail to eliminate basal-like, CSC-like and EMT cells. Inhibition of GLI1, although pharmacologically more challenging, could be preferable for the treatment of TNBC and to prevent metastasis and recurrence.

#### 4.10.2. GLI1-Induced Metastasis Through CXCR4

BC spreads preferentially to the brain, lungs, bones and liver. Metastasis to these organs accounts for the mortality associated to BC and worsens the morbidity of the advanced cases. To elucidate the role of GLI1 in BC metastasis, 4T1 cells stably expressing a luciferase reporter were transplanted into Balb/c mice, a model of lung BC metastasis. Overexpression of GLI1 increased the number of metastatic *foci* in the lungs, whereas inhibition of GLI1 activity with GANT61 reduced the number of metastasis [167].

Selective spreading of BC is believed to occur through the existence of a favorable niche in the target organs by secretion of high levels of the chemokine CXCL12 [168]. BC cells express high levels of the CXCL12 receptor CXCR4 [169] which, like SMO, belongs to the GPCR superfamily. The expression level of CXCR4 in BC correlates with poor prognosis, presence of distant metastasis and reduced overall survival of BC [170,171,172,173,174,175,176], and other cancer-types patients [172,173,174,175,176]. A second receptor for CXCL12, the CXCR7/ACKR3 protein is upregulated in cancer and is also believed to contribute to metastasis [177]. GLI1 increases metastatic potential by tuning up the levels of the CXCR4/CXCR7 receptors in BC cells and in other cancers [103,178,179,180,181,182]. Interestingly, GLI1 also induces LCP1/L-PLASTIN, a signaling mediator of CXCL12/CXCR4 signaling in BC cells, resulting in enhanced ERK phosphorylation and cell migration [183]. These results point to the therapeutic inhibition of GLI1 as a rational approach to reduce the metastatic burden in BC.

### 4.11. Hh Signaling and BC Microenvironment

The tumor microenvironment or stroma plays important roles in tumor development and facilitates metastatic spread [184]. The stroma is composed by endothelial cells, immune cells, adipocytes, and activated fibroblasts, also known as “cancer-associated fibroblasts” (CAFs) [185]. CAFs fuel tumor cells by secreting soluble factors [186,187,188,189] that contribute to metastasis and chemoresistance by inducing extracellular acidification, inflammation, activation of matrix metalloproteases, and reducing the stability of chemotherapeutic drugs [186,190,191]. Intriguingly, some studies suggest that normal fibroblasts acquire a catabolic CAF phenotype when exposed to chemotherapy [144], characterized by stimulation of glycolysis, autophagy and secretion of pro-inflammatory mediators. This remodeling of the microenvironment activates an antioxidant response, Hh and interferon signaling that contributes to increase CSC numbers in ER-positive BC [144].

A hypoxic microenvironment also contributes to upregulation of GLI1. While the mechanisms are still under debate, HIF-1α can stimulate GLI1 expression independently of SMO in TNBC cells, perhaps through NF-κB [161]. In addition, HIF-1α can induce SHH expression in fibroblasts, which can contribute to GLI1 induction in hypoxia in a paracrine manner [192].

## 5. Correlation of Hh Markers with Clinical and Histopathological Parameters of BC Patients

Analysis of the correlations of markers of Hh pathway activation with clinical and histopathological parameters is fundamental for discovering prognostic markers and developing new therapeutic strategies. Moreover, the study of the dysregulation of the Hh signaling pathway in samples from affected patients would strongly validate its role in the onset and/or progression of BC.

To date, the available information about the relevance of Hh signaling in BC patients is incomplete. Most of the studies that will be described below do not consider the whole diversity of patients that are differently affected by the disease. Moreover, many of them describe only few parameters in small size samples that do not allow straightforward extrapolations to the whole population of BC patients. Also, the methodology used and the considerations for quantification of immunohistochemistry results varies among groups. However, conclusions arisen from these studies are strengthened by data obtained from fairly recently created public access, massive, and comprehensive databases such as The Cancer Genome Atlas (TGCA) repository (supported by National Cancer Institute (NCI), the National Human Genome Research at the National Institute of Health (NHGRI), and the Curtis dataset Curtis dataset [193].

### 5.1. Hh Signaling in Different BC Subtypes

Results about the association between BC subtypes and Hh are controversial (summary Table 1). In one group of reports, a positive correlation of SHH, DHH, PTCH and GLI1 expression levels with ER-positive (but not with HER2) BC samples was previously described [75,96,194,195]. GLI1 nuclear staining, a marker for Hh activation, was reported to be higher in ER-positive than in ER-negative BC specimens [111]. Moreover, GLI expression was positively correlated with pS2 and GREB1, two ER target genes [196].

In contrast, a large body of research found no correlation between GLI1 or SHH expression with HER2 or ER-positive BC subtypes. For example, few studies reported higher levels of GLI in HER2+ specimens compared to normal tissue and established a positive correlation of HER2 with GLI1 [197,198]. Also, while SHH expression was detected in HER2+ tumors, the authors could not derive any correlation between SHH and HER2 expression nor SHH and ER expression [79,88,194,197,198]. Other authors also reported a lack of association between GLI1 and ER [197,198] or HER2 [103,194], while others found an inverse correlation between GLI1 and ER [10,199]. Additionally, TNBC tumors showed higher expression of SMO and GLI1 in comparison to those in non-TNBC [10].

More recent results with larger populations sorted out the discrepancy. TNBC and luminal-B tumors were found to overexpress SHH, DHH, PTCH1, SMO and GLI1 in a cohort of Pakistani patients. No correlation of Hh molecules with HER2 status was observed. These results were confirmed by data obtained from the TGCA repository [4]. Moreover, high expression levels of members of the Hh pathway was associated with shorter survival related to occurrence of metastasis in patients with luminal-B tumors [4].

Enhanced expression of SHH was also associated with TNBC and basal-like BC phenotype [79,195]. In a TNBC cohort, increased SHH and SMO expression was associated with high histological grades, and SMO and GLI1 correlated with high tumor stages [10]. A significant association was also found between nuclear GLI1 staining and P-Cadherin (a basal-like phenotype marker) [199]

The level of GLI2 and PTCH1 in TNBC and basal-like BC patient’s samples was found to correlate with the expression of FOXC1. Analysis of TGCA and Curtis database [193] also reflected these correlations. In addition, there was a strong correlation between the Hh gene signature (composed of 13 genes related to Hh signaling) and decreased disease-specific survival [131].

### 5.2. Prognostic Value of Shh in BC

Higher levels of SHH protein expression is detectable in a significant fraction human BC tumors (IDC, DCIS, and DCIS with micro invasion) respect to non-neoplastic breast tissue, with higher percentage of positive patients in IDC compared to the rest [111] (Table 2). High levels of SHH were associated to poor prognosis and reduced overall survival [4,9,79,95,103,198,200] (Table 2). SHH and DHH overexpression was positively correlated with Ki-67 index, tumor stage and grade, lymph node involvement, metastasis, recurrence, and BC-specific death [4,10,79,195,197,198]. A significant correlation was also found between SHH expression and a lower tumor grade [197], suggesting that SHH signaling is relevant not only in late stages to promote progression and recurrence but also in early stages to enhance tumor growth and proliferation.

BC in young patients tends to be more aggressive and is associated to poor outcome [201]. Indeed, a recent study revealed that tumors from premenopausal women (patients < 45 yrs) overexpressed SHH, DHH and GLI1, while DHH was higher in postmenopausal patients [4]. Both SHH and DHH were correlated with early onset of disease [4]. However, a small size study performed mainly with postmenopausal women showed an association of SHH with postmenopausal status [195]. SHH is an independent predictor of age, ER expression, distant metastasis, and overall survival [4,195]. Hence, it would be important to determine the need of including Hh inhibitors to adjuvant therapy, for both young and post-menopausal BC patients.

A strong positive correlation was observed between SHH and GLI1 expression [88,103]. SHH levels were increased in inflammatory breast cancer (IBC) samples respect to non-IBC and its expression was predictive of relapse [202].

NF-κB binds to demethylated regions on the *SHH* promoter and enhances its transcription [95]. Hypomethylation of *SHH* promoter region was also observed in BC tumors with higher levels of SHH and NF-κB nuclear staining [88]. Those patients experienced poor outcome, however neither *SHH* methylation status nor NF-κB nuclear expression were prognostic of overall survival [95].

When IHH expression was also investigated in BC, it was found to be reduced in cancerous tissues, and no associations of IHH with clinical parameters were found [4,203].

#### Prognostic Significance of Serum SHH Levels in BC Patients

BC patients’ prognosis is based on histopathological characteristics of tumors such as tumor subtype, tumor size and grade, and lymph nodes metastasis. Even though hormone receptors are good as diagnostic and prognostic markers, they fail to predict long term progression or recurrence [204]. Cancer and stromal cells secrete many factors which can be measured in the blood and serve as biomarkers. Specifically, SHH is released by tumor cells and is detectable in serum and it was shown to correlate with higher risk of early relapse, compared to patients with low or null levels of serum SHH. Serum SHH levels are also a predictive marker of disease-free and overall survival [200].

### 5.3. PTCH1 and SMO Expression in BC Tumors

Hh signaling can be constitutively activated (independently to the presence of ligand) either by inactivating mutations in *PTCH1,* or by activating mutations in *SMO* [205]. Missense PTCH1 mutations were found in some BC patients [72], and a specific PTCH1 polymorphism was linked to the association between the risk of BC and the use of oral contraceptives [206]. No SMO mutations have been reported to date in BC.

Results about PTCH1 expression in tumors are contradictory and probably influenced by the poor quality of most commercial PTCH1 antibodies. A decrease in PTCH1 expression was observed in ductal carcinomas in situ (DCIS) and invasive ductal carcinomas compared to mammary hyperplasia and normal breast tissue [5,10,67,96] (Table 2). Also, a significant association was found between lower PTCH1 expression and low ER expression, with higher tumor grade and with absence of lymph node invasion [96]. High PTCH1 expression was associated with increased risk of recurrence in one study [198]. PTCH1 expression correlated with GLI1 and GLI2, and not with GLI3, as expected [96]. However, some other groups found similar levels of PTCH1 in IDC and normal breast [207], or elevated PTCH1 expression in BC samples but no expression in adjacent normal tissue [4,75,79,198]. In a recent study, PTCH1 expression was reported to be higher in tumors of postmenopausal patients [4] Low PTCH1 mRNA levels (compared to normal) but not protein levels were also reported [203]. Other studies concluded that PTCH1 expression has no prognostic significance or correlation with histopathological parameters in BC [67,79]. This data was supported by data obtained from datasets of Oncomine (TCGA breast and Finak breast). Elevated expression of PTCH1, which suggests activation of the canonical Hh pathway, was correlated with the Ki-67 index, and with shorter metastasis-free survival in luminal-B breast cancer patients [4]. It is relevant to remark that many of the results described above were obtained from immunostaining data, but only two of the studies [67,96] used well controlled, validated antibodies with larger sample sizes. This, at least in part, could explain the contradictory results. 

Expression of SMO was observed in DCIS and IBC but not in normal tissue, correlated with tumor size, lymph node involvement and increased risk of recurrence ([198] (Table 2), but not with histological grade or other clinically relevant markers. Higher expression of SMO was also reported in mammary hyperplasia [10]. SMO levels are not correlated with PTCH1 expression, neither in in DCIS or IBC [67]. This is not surprising because SMO levels are not regulated by the activation status of the Hh pathway. In TNBC, a correlation between SMO expression and histological grade and tumor stage was reported [10]. Riaz et al. found no association of SMO expression with patient’s age or metastasis, however it was associated with earlier onset of disease and with the TNBC subtype [4]. Altogether, these results highlight the possibility that targeting molecules downstream of SMO would result in more efficient therapies for BC patients.

### 5.4. GLI1 Expression in BC Tumors

GLI1 staining is undetectable or weak in normal breast tissue, but increases in mammary hyperplasia [10,199] (Table 1). GLI1 protein was overexpressed in different types of BC (IDC, DCIS, and DCIS with micro invasion) respect to normal breast tissue, with the highest percentage of nuclear GLI1 in non-neoplastic to IDC [4,75,111,197,198,199,203]. These findings suggest that Hh signaling was constitutively activated in most cases of IDC and in 30% of DCIS [111].

We have previously described (in 4.1) the controversies regarding the relationship between GLI1 and ER (as a marker of luminal subtype) (Table 1). In ER-positive BC, increased GLI1 expression correlated with early disease onset, increased SHH expression [111], high Ki-67 index [4], higher histological grade [4,10,197], advanced tumor stage [4,10,103], lymph node involvement [4,10,103], metastasis [4], and shorter overall survival and disease-free survival [98,103,199] (Table 2). Higher GLI1 expression was associated with lower distant metastasis-free survival (DMFS) in grade III patients and in ERα-positive BC patients [4,196], and with an increased risk of recurrence [198]. Elevated GLI1 expression was an independent predictor for age, ER expression, distant metastasis, and short overall survival [4,199], and disease-free survival [199]. GLI-1 did not correlate significantly with tumor size [197,198]. Elevated GLI1 was also observed in pre-menopausal BC [4].

These findings highlight the value of GLI1 not only as a target for therapy by also as a prognostic marker.

## 6. Targeting the Hh Pathway in BC: Past and Current Preclinical and Clinical Trials

The increasing knowledge of the central role of Hh signaling in cancer led to the development of pathway-specific inhibitors and to the repurposing of drugs that also interfere with Hh/GLI. The vast majority of Hh pathway inhibitors are SMO antagonists, given the druggability of GPCRs. Of those, Vismodegib and Sonidegib have been approved by the U.S. Food and Drug Administration (FDA) for the treatment of basal cell carcinoma (BCC) [209,210] and medulloblastoma [210] and Glasdegib was approved for the treatment of acute myeloid leukemia. In addition to SMO inhibitors, a monoclonal antibody that blocks SHH and IHH binding to PTCH1 is successfully used in research and, more recently, drugs that act to the level of the GLI transcription factors started to be investigated. Other compounds with high safety profile like the antifungal itraconazole [211], or that have been used in the clinic for many years, like arsenic trioxide and glucocorticoids, were repurposed to reduce GLI activation, but their mode of action is unclear. There are also a range of natural products with activity against the Hh pathway that are under investigation.

While the efficacy of SMO inhibitors in BCC and medulloblastoma is satisfactory, clinical trials in other solid cancers including colorectal, pancreatic, or lung cancer were disappointing [212,213,214]. The use of Hh inhibitors in BC has not been conspicuously researched, but some trials in phases I and II are still ongoing.

### 6.1. Monoclonal Anti-Hh Protein Antibody, 5E1 mAb

The 5E1 monoclonal antibody (mAb) prevents interaction of SHH and IHH with PTCH1 [215] by binding to a pseudo-active metalloprotease site of the Hh proteins in a Ca^2+^ and Zn^2+^-sensitive manner [215]. This binding surface overlaps with the interaction surface of HHIP, a natural Hh antagonist membrane protein that limits Hh signaling. By preventing the first step of the signaling pathway, it inhibits ligand-dependent canonical signaling and non-canonical signalling, but it is ineffective in the blockade of activation of the GLI proteins by crosstalk with other pathways. The 5E1 antibody has been used in numerous developmental biology, biochemical studies, and preclinical BC models. In one study, 5E1 mAb was shown to sequester Hh proteins secreted by BC cells and, as a consequence, reduce osteoclast differentiation [216]. Another study showed that intraperitoneal administration of 5E1 to mice with orthotopic implantation of BC cells significantly reduced tumor growth and metastasis to the lungs and liver, suggesting a therapeutic potential in BC [79,208]. Of note, humanization of 5E1 or development of similar antibodies for human use have not been yet announced.

### 6.2. SMO Inhibitors

Vismodegib and Sonidegib are the only SMO inhibitors approved for the treatment of advanced and metastatic BCC of the skin. BCCs arise by cell autonomous activation of the canonical Hh pathway, most commonly by loss-of-function of *PTCH1* (90%) and gain-of-function of *SMO* (almost 10%) [217]. Interestingly, the tumors tend to develop resistance to the inhibitors by novel mutations in SMO or by hijacking other pathways that activate GLI1 downstream of SMO, such as those described here in BC. Therefore, the utility of SMO inhibitors in BC might be limited to subtypes where activation of GLI1 is SMO-dependent, as appears to be the case in TNBC, or during EMT and metastasis, where paracrine activation of the stroma with Hh ligands seems to play an important role.

#### 6.2.1. Cyclopamine

Cyclopamine was the first described Hh pathway inhibitor. It is an alkaloid produced by corn lilies that poisoned the fetuses of pregnant ewes, resulting in newborn lambs with congenital defects similar to *Shh^−/−^* mice, including cyclopia. Cyclopamine binds to the hydrophobic core of SMO [218], and it has been used in a large number of in vitro studies as a selective SMO inhibitor with antitumor activity in a number of cancer types.

Several studies reported sensitivity of BC cell lines to cyclopamine; however most studies observe cytotoxic effects at concentrations much higher than needed to prevent GLI1 activation. Treatment of the breast epithelial cell lines MDA-MB-435, T47D, MDA-MB-231, MCF7 and normal epithelial MCF10AT cells with cyclopamine decreased the expression level of GLI1, but it selectively reduced viability and increased apoptosis in the cancer cells [102,203]. Cyclopamine induced cell cycle arrest in MCF-7 and MDA-MB-231 cells by inhibition of MAPK signaling and suppression of cyclin D1 expression, reduced invasiveness, and lowered NF-κB levels and metalloprotease secretion [219]. In addition, cyclopamine was found to sensitize BC cells to with standard chemotherapy, enhancing the antitumor effects of paclitaxel [220], EGFR inhibitors (afatinib and gefitinib) and tamoxifen [221,222] in xenografts animal models. In TNBC MDA-MB-231 cells, cyclopamine was shown to reduced cyclin D1 and BCL2, two classical GLI-target genes and, as consequence, to decrease proliferation and stimulate apoptosis [223]. Moreover, the treatment caused an increase in E-Cadherin and reduction of MMP2 and MMP9, limiting migration and invasion.

While cyclopamine was shown to be effective in vitro and in some animal studies, the dosage used is in most cases much higher than needed to block SMO, suggesting the contribution of nonspecific cytotoxic effects [224]. This notion is farther supported by the low levels of SMO expression in many BC cell lines in which cyclopamine shows cytotoxicity. There is no real justification on the dosages chosen for most BC studies [97,102,219]. In addition, the pharmacokinetic characteristics of cyclopamine make it a poor therapeutic choice [225], which has led to the development of derivatives with better profile. In particular D-homocyclopamine, cyclopamine-4-en-3-one, and 3-keto-*N*-aminoethyl aminocaproyl digyrocinnamoyl (KAAD)-cyclopamine, with better solubility and stability than cyclopamine, have shown anticancer properties [226].

#### 6.2.2. GDC-0449 (Vismodegib)

GDC-0449, a synthetic SMO inhibitor with good pharmacokinetic and pharmacodynamic profile, has entered clinical trials as a component of combination therapy for TNBC, but its efficacy is still unclear. In one trial, GDC-0449 was used in combination with a NOTCH pathway inhibitor, RO4929097 (trial ID NCT01071564) (Table 3). Since NOTCH and Hh are involved in self-renewal of stem cells, the rationale was that inhibiting both pathways would eliminate CSCs and prevent recurrence in advanced cases of TNBC. Unfortunately, the trial was suspended after the report of life threatening arrhythmic episodes.

Another phase II trial is still recruiting TNBC patient to study the safety and efficacy of GDC-0449 and standard neoadjuvant chemotherapy vs. standard chemotherapy alone (trial ID NCT02694224) (Table 3). The results are not yet available.

#### 6.2.3. LDE225 (Erismodegib, Sonidegib)

LDE225 has shown an acceptable safety profile and was approved in 2015 by the FDA for the treatment of locally advanced or metastatic BCC in adult patients. It shows good efficacy in BCC and relapsed medulloblastoma. Its antitumor activity against medulloblastoma was associated with reduction of GLI1 mRNA levels [227]. In addition, LDE225 promoted disease stabilization in lung adenocarcinoma, spindle cell sarcoma and BC [228]. In in vitro and in vivo studies, LDE225 showed efficacy against melanoma [229].

Safety evaluation of combination therapy of Sonidegib and paclitaxel in a phase I trial of advanced solid tumors demonstrated good tolerability [230] (trial ID NCT01954355) (Table 3). The combination showed antitumor activity against ovarian, anal and BC; however, it was not associated with modulation of canonical Hh signaling, as determined by IHC in archival specimens.

LDE225 was also evaluated in combination with docetaxel in a phase Ib trial in advanced TNBC patients to determine the maximal tolerated dose and recommended phase II dosage [231] (trial ID NCT02027376) (Table 3). The study revealed good tolerability of the combination therapy but the small number of patients recruited preclude prediction of its antitumor efficacy. Nonetheless, the investigators reported one case of complete response and two cases of disease stabilization, supporting the need of larger studies. Another phase Ib clinical trial set out to determine the maximal dose of LDE225 and BKM210, a PI3K inhibitor, in patients with metastatic BC, advanced pancreatic cancer, metastatic colorectal cancer and glioblastoma multiforme has been completed but the results are still unpublished (trial ID NCT01576666) (Table 3).

#### 6.2.4. Itraconazole

Itraconazole, a broad-spectrum anti-fungal triazole agent, has been repurposed as an inhibitor of the Hh pathway in a screen of drugs previously tested in human that inhibit the canonical Hh signaling [211]. Itraconazole interferes with fungal sterol biosynthesis by inhibition of lanosterol 14-α-demethylase [232,233]. However, its inhibitory effect on Hh signaling is distinct and appears to be through prevention of ciliary accumulation of SMO. When administered systemically in mice, itraconazole suppressed the growth of medulloblastoma and reduced Hh activation markers at similar concentrations than required for its antifungal activity [211]. In the BC cell lines MCF-7 and SKBR-3, itraconazole induced autophagic cell death and G0/G1 cell cycle arrest [234]. In combination with 5-fluorouracil, it further reduced MCF-7 cell viability and proliferation [235]. In addition to its direct antitumor activity, itraconazole was shown to reduce angiogenesis in vitro and in vivo by affecting lanosterol 14-α-demethylase [236]. In effect, a pilot trial in 13 metastatic BC patients showed that itraconazole decreased the plasma levels of βFGF and PIGF, two proangiogenic factors, and increased the level of thrombospondin-1, an inhibitor of angiogenesis [237] (trial ID NCT00798135,) (Table 3). Thereby, part of its growth inhibitory action in solid tumors could be due to its antiangiogenic properties.

Itraconazole has also been used as a P-glycoprotein inhibitor in a drug-drug interaction study with another SMO inhibitor, vismodegib (trial ID NCT01772290) (Table 3). The results showed that they can be administered simultaneously without the risk of significant pharmacokinetic interaction [238].

Finally, the low cost of itraconazole and well known safety profile makes it a possible drug of choice for use as an adjuvant in cancer treatment in developing countries or areas of socioeconomic disadvantage. 

### 6.3. GLI Antagonists

A number of inhibitors of that act at the level of the GLI transcription factors have been discovered and tested in preclinical models. A few have been described to directly bind to GLI (GANT61, Glabrescione B (GlaB)) [240,241], but in most cases there is not clear mechanism of action. Some inhibitors seem rather specific for Hh/GLI signaling, like GANT61, GANT58, GlaB, and the Hh Pathway Inhibitors 1-4 (HPI 1-4). Others prevent GLI activation secondary to interfering with other cellular pathways or processes. These include arsenic trioxide (ATO), arcyriaflavin, staurosporine, HDAC1/2 inhibitors, physalin B/F, pyrvinium, I-BET151, JQ1, and some natural products. Here, we will describe the known or probable mechanisms of action and their efficacy in cancer.

#### 6.3.1. Hh Pathway “Specific” GLI Inhibitors

The main known function of this group of compounds is to antagonize the GLI transcription factors by preventing DNA binding, ciliary trafficking, or processing. They are classified as GLI antagonists because they act downstream of SMO and SUFU, a cytoplasmic negative regulator of GLI, and can reduce the transcriptional activity of overexpressed GLI1 and/or GLI2. GANT61 and GANT58 were the first identified compounds fulfilling these characteristics [242]. GANT61 and GlaB have been shown to bind to the Zn-finger motifs of GLI1 and GLI2 and as a result interfere with binding of GLIs to DNA [240,241,242]. The HPI compound series, which are structurally unrelated, inhibit GLI function by diverse mechanisms: HPI-1 blocks GLI ciliary trafficking and nuclear entry, HPI-2 and HIP-3 block maximal activation of full length GLI1 and GLI2 by an unknown kinase, and HPI-4 prevents GLI activation by interfering with primary cilium assembly [243]. Reports of use of GLI inhibitors in BC are limited to GANT61 and GANT58 [100,131,132,197]. GANT61 showed antiproliferative activity, induced apoptosis and reduced expression of mesenchymal markers GLI1-positive TNBC cells, and reduced xenograft growth in HER2^+^ cells. Despite being promising as GLI1/2 inhibitor, GANT61 is poorly stable under physiological conditions and will require optimization before it can be used in vivo [244].

A recent study compared the effect of GANT58 and HIP-1 in breast cancer cells. The results indicate that HPI-1 is more effective than GANT58 (which has a high IC_50_) in inducing apoptosis, reducing proliferation, decreasing invasion and reducing the percentage of CSCs in the cell population [245]. GANT61 has been evaluated in numerous preclinical studies, due to its better inhibitory capacity and lower IC_50_ than GANT58. GANT61 has been shown to target all the hallmarks of cancer, including proliferation, viability, apoptosis, autophagy, DNA repair, EMT, stemness and immune response in different cancer types, including BC [246]. GANT61 was shown to inhibit proliferation of many BC cell lines, including those expressing high levels of GLI1 (MDA-MB-231 and MCF-7), and irrespective of the ER-status [4,247]. In ER-positive BC cells, estrogen increases the CSCs fraction, apparently by a non-canonical upregulation of GLI1 and GLI2. GANT61 not only inhibited proliferation of ER-positive cells, but also reduced the CSC compartment in combination with antiestrogens. Inhibition of GLI-mediated responses decreased invasion and migration, and increased apoptosis by downregulation of BCL2 and upregulation of BAX [197].

In TNBC cell lines, believed to contain a larger CSC population than ER-positive BC, administration of GANT61 decreased proliferation and increased apoptosis [248]. The effect of GANT61 was skewed towards the CSC population, while paclitaxel inhibited proliferation mostly in non CSC cells. The combination of both compounds additively reduced cell growth and CSC numbers, suggesting that this combination has potential for the treatment of TNBC.

#### 6.3.2. Indirect GLI Inhibitors

The drugs in this category inhibit GLI transcriptional activity indirectly by interfering with primary cilium formation, trafficking of GLI proteins into the cilium, regulation of post-translational modifications of GLIs and by epigenetic silencing. For example, forskolin and pyrvinium stimulate GLI1 and GLI2 phosphorylation by PKA and CK1α, inducing their degradation [249,250]. Staurosporine and physalins B/F reduce non-canonical activation of GLI1 and GLI2 by the PKC-*δ*/MAPK axis [44], resulting in inhibition of ligand-independent GLI activity [251]. HDAC1/2 inhibitors block deacetylation of GLI1 and GLI2, reducing their maximal activity [252]. Inhibitors of the Bromo and Extra C-terminal (BET) bromodomain protein (JQ1 and I-BET151) result in epigenetic silencing of GLI1 and GLI2 [253,254].

Arsenic trioxide (ATO) was shown to prevent ciliary accumulation of GLI2 and to reduce its stability and transcriptional activity [255]. ATO is a chemotherapeutic agent commonly used as first-line treatment for acute promyelocytic leukemia (AML) because of its ability to induce apoptosis of leukemia cells [256]. ATO also induces apoptosis in many solid cancers, including BC and in in BC cell lines [256,257]. While the mechanism of action of ATO toxicity remained a mystery for many years, it was recently shown that the protein PIN1 is the molecular target of ATO in AML [258]. However, whether PIN1 mediates the inhibitory effect of GLI activation has not been investigated. ATO also blocked Hh pathway activation in cell and animal models of Ewing’s sarcoma, suggesting that its anticancer activity might be partly due to targeting the Hh pathway [259]. A phase II trial of ATO in women with locally advanced and metastatic BC (trial ID NCT00075413) (Table 3) was terminated by difficulties with patient recruitment.

### 6.4. SMO Inhibitors vs. GLI Antagonists

Direct comparison of the effectiveness of SMO vs. GLI antagonists in a large number of BC cell lines (MCF-7, T47-D, MDA-MB-231, MDA-MB-468, MDA-MB-453, BT-474, and SK-BR-3, and mouse TUBO) showed that GANT61 was superior to GDC-0449 [197]. However, both types of Hh inhibitors showed target engagement (reduced Hh signaling), inhibited BC cells survival, induced apoptosis, reduced p21-Ras and MAPK signaling, and reduced nuclear localization of NF-kB. Both drugs also suppressed tumor growth of TUBO cells xenografts in BALB/c mice to a different extent. This study suggests that targeting the Hh pathway downstream of SMO is a more effective strategy to treat BC.

### 6.5. Phytochemicals Targeting HH

A growing number of plant-derived bioactive compounds, and their synthetic derivatives, have shown anticancer activity by targeting cell proliferation, survival, migration, EMT, and other essential processes in cancer cells [260]. Many natural compounds show activity against WNT [261], NOTCH [262], NF-κB [263], PI3K/Akt/mTOR [264], MAPK [265], and Hh [6] pathways in BCs [266]. Important cancer drugs like paclitaxel, camptothecin, vinblastine, vincristine, topotecan were optimized from natural compounds [267].

#### 6.5.1. Genistein

This isoflavone, present in soy and other plants, has shown activity against various cancers, particularly BC and prostate cancer. Genistein has been well-characterized as a nonspecific tyrosine kinase inhibitor, but it also affects many signaling pathways and processes [268,269]. Genistein has been reported to reduce MCF-7 cells proliferation, induce apoptosis, and reduce the number of mammospheres by suppressing the CSC population [270]. Accordingly, genistein suppressed tumor growth in BC xenografts. Interestingly, it was shown to reduce SMO and GLI1 expression both in vitro and in vivo, which can contribute to the reduction in CSC numbers [270].

#### 6.5.2. Pterostilbene

Pterostilbene (PTE) is a resveratrol analog present in blueberries and grapes with bactericidal activity [271]. Testing of PTE in MCF-7 BC cells showed that it is selectively cytotoxic toward the CSC population, reducing mammosphere formation by inducing necrosis of CSCs [272]. Additionally, PTE potentiated the efficacy of paclitaxel and inhibited the expression of SHH [272]. Further investigation is needed to define pterostilbene as a good candidate for BC treatment.

#### 6.5.3. Curcumin

The medicinal properties of curcumin, a phenolic compound that accounts for 2-6% of the turmeric spice, has been well documented in the Ayurveda [273]. Numerous studies have shown curcumin has biological activity against inflammation, ischemia, cancer and aging [274,275,276]. The Hh signaling pathway is one of many molecular pathways affected by curcumin [277,278,279]. The literature shows that WNT/β-catenin, NOTCH, NF-κB, PI3K/AKT/mTOR, and MAPK are also targets of curcumin [280]. The lack of a selective target and the rapid metabolism of curcumin in humans make it a poor candidate for therapeutic development. Improved delivery of curcumin in nano-micelles or as conjugate of a self-assembling variant of human apoferritin improved its solubility and stability and displayed cytotoxic effects on MDA-MB-468 and MDA-MB-231 cells by inducing G0/G1 cell cycle arrest and reduction of PI3K/Akt signaling [281], enhanced the cytotoxic effect of doxorubicin by interfering with multidrug resistance transporters [281], and reduced TNBC tumor growth in mice without systemic toxicity [282].

In vitro studies showed that curcumin downregulated the expression of SHH, SMO, GLI1 and GLI2 in mammosphere forming cells from MCF7 and SUM159 cells [283]. However, stimulation of the Hh pathway with the SMO agonist purmorphamine or by overexpression of GLI1 increased CSCs markers and suppressed the effect of curcumin [283].

Epidemiological studies support a reduction of BC risk by the consumption of certain protective phytochemicals, minerals, and anti-oxidants [284,285,286,287,288]. In fact, at the moment, two clinical trials are including curcumin as a dietary supplement. Even though formal results are not yet available, these studies will help determine the benefits of curcumin for BC patients and high-risk women.

#### 6.5.4. Resveratrol

Resveratrol has been intensively studied as an antioxidant compound in vitro and in clinical trials in different cancers [289,290,291,292]. Resveratrol is a natural non-flavonoid polyphenolic compound. Like curcumin, it has been reported to act on many targets, mostly indirectly, including AMPK, SIRT1, COX1 and COX2, PDEs, and the NF-kB, PI3K/Akt, MAPK and Hh signaling pathways [291,292,293,294]. Some studies reported that resveratrol could elicit antitumor effects on BC cells by affecting proliferation, suppressing anti-apoptotic pathways, inhibiting EMT, sensitizing to chemotherapeutic compounds and reducing multidrug resistance [295,296]. In some animal studies, resveratrol was found to suppress mammary gland differentiation and proliferation, reduce lipid peroxidation, angiogenesis, tumor growth and metastasis [295,296]. However, other studies reported adverse effects, at least in some types of BC, due to increased HER2 expression and activation of mTOR, supporting HER2-positive and ER-positive BC growth [297]. Resveratrol also reduced TNBC susceptibility to paclitaxel by interfering with paclitaxel oxidative stress-induced DNA damage and increasing Cyclin B/CDK1 [298,299,300]. Since a biphasic effect of resveratrol was reported in vitro, its effects on BC cancer cells might be dose-dependent [301,302,303].

Administration of resveratrol together with curcumin was shown to increase apoptosis and reduce the expression of SHH, SMO, GLI1, C-MYC, Cyclin-D1 while upregulating p21^Waf/Cip1^ in MCF-10A cells in vivo and in xenografts in vivo [304]. However, its antioxidant effect appears to reduce chemotherapy efficacy and therefore, its use in BC should be carefully considered.

#### 6.5.5. Nitidine Chloride

Nitidine chloride (NC) is a natural alkaloid with anticancer activities in BC cells, in particular in combination with doxorubicin [305]. NC was reported to reduce proliferation and sphere formation capacity of MDA-MB-468 and MCF-7 cells and to downregulate SMO, PTCH1, GLI1 and GLI2 expression. In combination with cyclopamine, NC enhanced its inhibitory effect on BC cell migration, upregulated E-Cadherin and downregulated N-Cadherin and Vimentin expression [306]. Thus, the combination of SMO inhibitors and NC could reduce EMT and CSC-like properties in BC.

#### 6.5.6. Metformin

Metformin, used in the treatment of type-2 diabetes, was shown to reduce the risk of some cancers by inhibiting Hh signaling, including BC [307,308,309,310,311,312]. In vitro, metformin was reported to selectively kill CSCs and reduce migration and invasion, concomitantly with downregulating of several proteins of the Hh pathway [313,314]. Metformin also impaired SHH-induced proliferation, migration and invasion of BC cells and reduced tumor growth in vivo [307]. Interestingly, the inhibitory effect of metformin on Hh signaling is independent of its role in AMPK regulation. Moreover, the high safety profile of metformin supports its evaluation in clinical trials in BC.

### 6.6. Alternative Strategies to Target Hh Pathway in BC

#### 6.6.1. Statins

Cholesterol is an essential regulator of the Hh signaling pathway. Cholesterol is mobilized by the transporter function of PTCH1 [315], it is essential for SMO activation [316,317,318], and is necessary for Hh proteins processing into active signaling molecules [319]. Derivatives of cholesterol, like 25-hydroxysterol (OHC), 27-OHC and 20(S)-OHC, are potent endogenous agonists of SMO [320,321,322,323]. Early experiments showed that interference of cholesterol biosynthesis by inhibition of 3-hydroxy-3-methylglutaryl-coenzyme A reductase (HMGCR), which converts HMG-CoA to mevalonate, abolishes Hh signaling in cells. HMGCR is the target of statins, compounds used to lower cholesterol levels in patients with high risk of cardiovascular events.

A recent study showed that metabolic formation of 27-OHC by CYP27A1 increased BC tumor growth and metastasis in mice by acting as agonist of both ER and Liver X receptor (LXR) nuclear receptors, and that it correlates with tumor grade in human specimens [324]. However, it was not investigated if 27-OHC effects were also mediated by SMO. The diet-containing oxysterols 25-OHC, 27-OHC, 22-OHC and 7-ketoC (which is metabolized in tissues including breast into 7-keto-25-OHC and 7-keto-27-OHC) are strong agonists of SMO, binding to its cysteine-rich extracellular domain (CRD) with submicromolar EC_50_s, a range achievable by a normal Western diet. It is possible, then, that the upregulation of SMO observed in 70% of ductal carcinomas and 30% of TNBCs [67], makes those tumors sensitive to Hh pathway activation by dietary oxysterols.

There is strong evidence that statins have a preventative effect in ER-positive and ER-negative BC, both in cancer cell lines and epidemiological studies [325,326,327,328,329,330,331,332,333]. Nonetheless, some large studies do not support a beneficial effect in BC [330,334,335,336,337,338,339,340], although some authors argue that those studies do not differentiate between lipophilic and hydrophilic statins, which could account for the lack of effect [341,342]. A number of short-term single-arm intervention studies investigating the effect of statins on BC biomarkers have produced disappointing results [343,344,345]. In one multi-site study of high-risk premenopausal women to investigate the effect of atorvastatin on mammographic density (MD) and serum IGF-1 levels, both associated with increased risk of BC, found that this lipophilic statin did not reduce MD and indeed increased IGF-1, despite reducing serum cholesterol [239] (NCT00914017) (Table 3). While the increase of IGF-1 was unexpected, it was later discovered that IGF-1 are associated with HDL levels, and are inversely correlated to total cholesterol and LDL levels [346,347]. A short course of simvastatin (24 to 28 weeks) in 45 women with stage 0 to III breast cancer did not show any reduction in the MD of the contralateral breast [344]. A 6-month course of lovastatin in 26 women at high risk of developing BC also failed to show any improvement in the BC risk biomarkers [343]. Despite the apparent lack of effect when considering MD as primary endpoint, fluvastatin treatment in stage 0 to 1 BC reduced Ki-67 and increase cleaved caspase-3, suggesting a reduction of proliferation and an increase of apoptosis, after only 3–6 weeks of treatment [345].

Because of these findings, it has been suggested that future trials should focus on markers of proliferation, apoptosis and inflammation, although MD remains one of the most precise indexes of BC risk.

#### 6.6.2. Vitamin D

BC patients with high expression of the vitamin D receptor (VDR) have reduced incidence of metastasis [348] and better survival than those with low VDR [349]. In addition, some VDR polymorphisms were associated with higher risk of BC [348,350]. The protective role of the VDR is supported by the finding of a higher proliferative state in normal mammary glands of VDR-deficient mice compared to wild type counterparts [351]. The mammary glands of *VDR^−/−^* animals were more undifferentiated instead of exhibiting the normal ductal structures. Moreover, *VDR^−/−^* mice were more sensitive to chemical carcinogens [352].

The VDR binds to the vitamin D metabolite 1α,25-dihydroxyvitamin D_3_ (1,25D_3_) and dimerizes with the 9-cis-retinoic acid receptor (RXRα) to stimulate expression of VDR-responsive genes [353]. Addition of 1,25D_3_ to BC cells reduced proliferation and induced apoptosis and differentiation [354]. 1,25D_3_ was also shown to downregulate miR-214 expression, a microRNA that targets SUFU, a negative regulator of the Hh pathway [354].

Over 10 years ago, it was proposed that PTCH1 could inhibit SMO in a non-cell autonomous manner through the transport of Pro-vitamin D3 [355]. However, genetic analysis of mammalian, avian, and *Drosophila* embryos strongly argue in favor of a strict cell-autonomous effect of PTCH1, and are supported by recent biochemical studies [315]. Nevertheless, vitamin D shows a weak inhibitory effect on SMO [355], suggesting that part of its anticancer effects could be mediated by inhibition of Hh signaling.

A phase 2 “window” trial of short term effects of vitamin D administration in BC patients awaiting surgery was completed but the results were not published (trial ID NCT01948128) (Table 3). In addition, a phase III trial testing the effect of Vitamin D3 on proliferation and apoptosis markers is ongoing (trial ID NCT01608451) (Table 3).

## 7. HH Signaling and Resistance Mechanisms in BC

### 7.1. The Hh Pathway and Tamoxifen Resistance

Tamoxifen is a well-established antiestrogen used on adjuvant therapy in ER positive BC patients. However, the success of this drug is hampered by the development of resistance. Therefore, it is crucial to identify the mechanisms involved in tamoxifen resistance and to design new strategies of treatment for these patients. The role of Hh signaling in the acquisition of resistance is not clear, but it is essential to determine its significance as a target for combinatorial treatment of BC patients.

Key factors involved in tamoxifen resistance are loss of ERα function [356,357,358], ERα mutations [359,360,361], and the activation of ER signaling through the dysregulation of tyrosine kinase receptors (EGFR, HER2, IGF1R, FGFR), and a number of signaling pathways, including PI3K-PTEN/AKT/mTOR and NF-κB, among others [362,363].

Tamoxifen modifies the expression of components of the Hh pathway. GLI1 expression and cell proliferation increase upon tamoxifen treatment of both ERα-positive/HER2-negative and ERα-positive/HER2-positive cell lines [364]. The expression of PTCH1, GLI1 and its target genes (*SNAIL*, *BMI1* and *MYC)* is higher in tamoxifen-resistant compared to tamoxifen-sensitive cells [98,196]. MYC and MBI1 protein levels were positively correlated to tamoxifen resistance [98]. Tamoxifen-resistant cells, when transplanted in mice, form aggressive metastatic tumors with elevated expression of Hh signaling markers [98]. Moreover, the blockage of ERα signaling caused by tamoxifen is bypassed by Hh activation through the PI3K/Akt pathway [98]. Tamoxifen-resistant cell proliferation depends on HHAT (Hh acyltransferase), which catalyzes the palmitoylation of SHH [365,366,367]. GLI1 silencing was shown to attenuate cell proliferation and to enhance tamoxifen cytotoxicity in both tamoxifen-resistant and -sensitive BC cells [196]. GLI1 depletion also decreases ERα protein levels and ERα transcriptional activity. Furthermore, combined administration of tamoxifen and GANT61 reduced cell growth compared to the effect of each inhibitor alone, independently of estrogen stimulation [196].

Previous studies on tamoxifen and cyclopamine have indicated that these two drugs can counteract their individual effects [132,222]. Cyclopamine and tamoxifen decrease the viability on ER positive BC cells when administered individually. However, simultaneous administration increased short-term survival and enhanced migration of the cells, along with upregulation of Hh-GLI signaling [222]. Additionally, it was also described that OPN, a secreted phosphoglycoprotein that is abundant in the tumor microenvironment, induces GLI1 in BC cells through a non-canonical ligand-independent pathway. As a result, cells acquire a mesenchymal phenotype and become resistant to cyclopamine [132]. Finally, ERα can be activated by SHH as part of a non-canonical GLI independent signaling pathway [222]

Altogether, these results suggest that tamoxifen resistance could be bypassed by the blockage of the Hh pathway. Inhibitors acting downstream of SMO, at the level of GLI1, are promising [247]. However, SMO inhibitors would regain relevance when non canonical GLI-independent pathways are activated, as those that promote angiogenesis and cytoskeletal reorganization [81,82].

### 7.2. The Hh Pathway and Chemoresistance in TNBC

Chemoresistance of TNBC has also been associated with the Hh pathway. GLI1 mediates resistance of TNBC cells to paclitaxel, doxorubicin, and cisplatin through the upregulation of two transcriptional targets: multidrug resistance protein 1 (MDR1) and breast cancer resistance protein (BCRP) [132]. The increase in MDR proteins could also explain the increased survival and proliferation of BC cells, which is also mediated by Hh signaling activation, after taxane treatment [368].

## 8. Conclusions

The evidence reviewed here highlights the relevance of the Hh signaling pathway in BC. Although more research remains to be done to determine the specific mechanisms of action of each member of the Hh pathway and its crosstalk with other signaling cascades, the data obtained to date suggest that dysregulation of this pathway is key for BC progression and metastasis.

**Figure 1:**
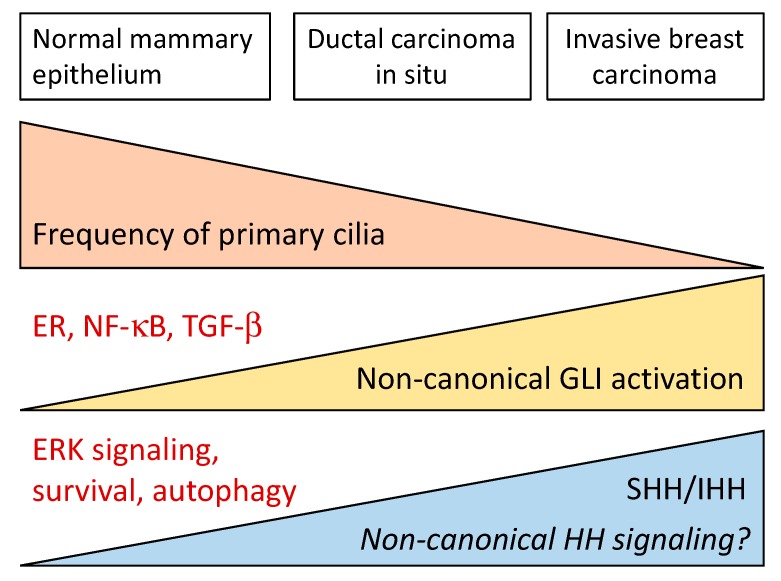
Proposed interpretation of the role of Hh signaling in BC from the combined pre-clinical and clinical data. As BC increases in grade and becomes more metastatic, the frequency of ciliated cells declines while GLI1 and Hh ligands expression increases. We postulate that the Hh ligands act by non-canonical signaling, increasing ERK signaling, inhibiting an apoptotic function of PTCH1 and promoting autophagy. GLI1 upregulation is presumably the result of crosstalk with other pathways (ER, NF-kB and TGF signaling).

As we discussed previously, upregulation of GLI1 is common in BC compared to normal mammary tissue and correlates with tumor grade, invasiveness and metastasis. However, it seems that GLI1 is upregulated mostly by crosstalk with other pathways aberrantly activated in BC rather than by Hh ligands, which are also upregulated in BC (Figure 1). Upregulation of Hh ligands might contribute to BC progression by modulation of non-canonical Hh signaling in epithelial cells and/or promoting a pro-oncogenic stroma. This conclusion is supported by the observation of a strong reduction of primary cilia frequency in all BC subtypes compared to normal tissue and by an abnormal elongation of the few remaining cilia [128]. Loss of functional cilia prevents Hh and SMO-dependent activation of GLI transcription. Nonetheless, cilia are also necessary for formation of the repressor forms of GLI2 and GLI3; therefore, loss of cilia potentiates non-canonical activation of GLI1 by reducing the amount of GLI2-R and GLI3-R [369]. If activation of GLI1 in BC occurs by SMO- and cilium-independent pathways, the prediction is that pharmacological targeting of SMO with FDA-approve inhibitors will fail to prevent disease progression while targeting of GLI1 and GLI2 might be preferable.

Further trials of Hh pathway inhibitors for treatment of BC patients belonging to TNBC, basal-like and luminal subtypes are needed, even if the expression of members of the Hh signaling pathways cannot be yet used as biomarkers, based on the contradictory evidence described. Most of the reasons of this discrepancy were here discussed, with one main concern being the small size of some of the cohorts studied. We also speculate that, due to the high heterogeneity among BC patients which has not been thoroughly considered, some results were masked or misleading. Crucially, future analysis of samples from patients should contemplate the BC intrinsic molecular subtypes which differ in their prognosis and responses to chemo- and hormonal therapy [370]. Nonetheless, the relevance of Hh signaling in BC was doubtlessly established as a marker of poor prognosis.

## Figures and Tables

**Table 1:** Association of members of the Hh pathway with BC subtypes.

	SHH	DHH	PTCH	SMO	GLI1
**ER+**	Overexpressed [4] Positive correlation [4] No correlation [79,88,194,197,198]	Overexpressed [4] Positive correlation [4]	Overexpressed [4] Positive correlation [4]	n.d.	Overexpressed [4] Positive correlation [4,75,194,195,196] High % nuclear staining [102,111] Negative correlation [10,199] No correlation [103,197,198]
**HER2+**	Overexpressed [197] No correlation [197] No correlation [4,79,88,194,195]	No correlation [4,75,194]	No correlation [75,194,195]	n.d.	Overexpressed [197] Positive correlation [197,198] No correlation [4,75,103,194,195]
**TNBC**	Overexpressed [4,79,195]	Overexpressed [4]	Overexpressed [4]	Overexpressed [4,10] Increased (respect to ER+ and HER2+) [10]	Overexpressed [4,10,102,199] Increased (respect to ER+ and HER2+) [10] High % nuclear staining [199]

**ER+**, estrogen receptor-positive BC; **HER2+,** HER2-positive BC; **TNBC**, triple-negative BC, **SHH**, Sonic Hedgehog; **DHH**, Deset Hedgehog; **PTCH-1**, patched homolog-1; **SMO**, smoothened; **GLI-1**, glioma-associated oncogene homolog-1; **n.d.**, non-determined.

**Table 2:** Correlation of members of the Hh pathway with clinical and histo-patological parameters of BC patients. .

		SHH	DHH	PTCH1	SMO	GLI1
**Expression**	**Non neoplastic**	Low [10,88,203,208]	Low [203]	Low [4,10,67,75,96]	Low or absent [10,67,198]	Low or Absent [4,10,198,199,203]
	**DCIS**	Increased * [10,88,111]	Increased * [4]	Low or absent [10,67,96] Increased * [4,75,198]	Increased * [10,67,198]	Increased * [4,10,75,88,111,198,199]
	**IDC**	Increased * [10,79,88,111]	Increased * [4]	Low or absent [10,67,96] Increased * [4,75,79,198]	Increased * [10,67,198]	Increased * [10,88,111,198,199]
**Correlations**	**ER**	Positive [195] None [3,9,12,14,15]	Positive [75,194,195])	Positive [96] None [198]	None [198]	Positive [75,194,195,196] None [103,197,198] Negative [10,199]
	**HER2**	None [3,8,12,14,15]	None [7,8,15]	None [75,194,195]	n.d.	Positive [4,9,14] None [8,15,17]
	**Ki-67**	Positive [4,79,195]	Positive [4]	Positive [4]	n.d.	Positive [4] None [199]
	**Age**	Positive [4] None [88]	Positive [4]	n.d.	Positive [4] None [4])	Positive [4] None [199]
	**Histological type**	Positive [88]	n.d.	n.d.	n.d.	None [199]
	**Histological grade**	Positive [4,10,79,195,197]	Positive [4]	Positive [67]	Positive [10,67]	Positive [4,197] None [199]
	**Tumor size**	Positive [4,79,198] None [88]	Positive [4]	Positive [198] Negative [96]	Positive [198]	Positive [4] None [197,198]
	**Early disease onset**	Positive [4]	Positive [4]	n.d.	Positive [4]	Positive [4]
	**Stage**	Positive [4,88,195]	Positive [4]	n.d.	Positive [10]	Positive [10,103] None [199]
	**Lymph node involvement**	Positive [4,200] None [88]	Positive [4]	Negative [96]	Positive [198]	Positive [4,10,103] None [197]
	**Distant metastasis**	Positive [4,79,195]	Positive [4]	n.d.	None [4])	Positive [8,16]
	**Pre-menopause**	Positive [4]	Positive [4]	n.d.	n.d.	Positive [4]
	**Post-menopause**	Positive [195]	n.d.	Positive [4]	n.d.	n.d.
	**Poor prognosis**	Positive [8,9,12,13,19,20,21]	Positive [4]	Positive [4]	Positive [4]	Positive [4,98,103,199]
	**Recurrence**	Positive [198,200,202]	Positive [198]	Positive [198]	Positive [198]	Positive [198,199] None [103]
**Independent predictor**	ER, age, DM, OS [4,195]	ER, age, DM, mortality, OS [4,195]	Age, mortality [4]	n.d.	ER, age, DM, OS [4]	ER, mortality [4]

**ER**, estrogen receptor; **HER2**, human epidermal growth factor receptor 2; **SHH**, Sonic Hedgehog; **DHH**, Deset Hedgehog; **PTCH-1**, patched homolog-1; **SMO**, smoothened; **GLI-1**, glioma-associated oncogene homolog-1; **DM**, distant metastasis; **OS**, overal survival; **n.d.**, non-determined. * Increased respect to normal breast tissue.

**Table 3:** Current clinical trials in breast cancer with drugs that target (directly or indirectly) the Hedgehog signaling pathway (Data from www.clinicaltrials.gov.).

Treatment	Target	Location	Setting	Clinical Status	Status	Number of Patients	Trial Identifier	Reference
Vismodegib + RO4929097s	Smoothened Antagonist + Gamma Secretase Inhibitor	United States	Advanced BC (metastatic or unresectable)	Phase I	T	13	NCT01071564	No results posted
Neoadjuvant Vismodegib + Paclitaxel + Epirubicin + Cyclophosphamide	Smoothened Antagonist + antimicrotubule agent +Topoisomerase inhibitor + antineoplastic agent	Spain	TNBC	Phase II	R	40	NCT02694224	No results posted
Sonidegib (LDE225) + Paclitaxel	Smoothened Antagonist + antimicrotubule agent	Switzerland	Solid tumors	Phase I	C	2 BC out of 18	NCT01954355	[230]
Sonidegib (LDE225) + Docetaxel	Smoothened Antagonist + antimicrotubule agent	Spain	Advanced TNBC	Phase I	C	12	NCT02027376	[231]
Sonidegib (LDE225) + BKM120	Smoothened Antagonist + PI3K Inhibitor	Australia, Europe and United States	Solid tumors including TNBC and ER/PR+/Her2- metastatic BC	Phase Ib	C	120	NCT01576666	No results posted
Erismodegib (LDE225)	Smoothened Antagonist	United states	TNBC	Phase II	W (Poor accrual)	68	NCT01757327	No results posted
Itraconazole	Anti-angiogenesis and HH pathway inhibitor	United States	Metastatic BC	Pilot trial	C	13	NCT00798135	[237]
Vismodegib + Rabeprazole or Itraconazole or Fluconazole	Smoothened Antagonist + proton pump inhibitor or antifungal drug	United States	Healthy volunteer	Phase I	C	92	NCT01772290	[238]
Arsenic Trioxide	HH/Gli pathway inhibitor	United States	Advanced BC	Phase II	W (no subjects recruited)	0	NCT00075413	No results posted
Curcumin	NF-kB DNA binding Inhibitor	United States	BC	Phase II	C	3	NCT01740323	No results posted
Curcumin (DS) + Docetaxel	Dietary phytonutrient + antimicrotubule agent	France	Her2- advanced or metastatic BC	Phase II	T	42	NCT00852332	No results posted
Curcumin (iv) + Paclitaxel	Antiproliferative, anti-invasive, and antiangiogenic + antimicrotubule agent	Armenia	Advanced and Metastatic BC	Phase II	R	75	NCT03072992	No results posted
Curcumin (DS)	Anti-inflammatory effect	United States	obese women at high risk for breast cancer	Pilot trial	ANR	30	NCT01975363	No results posted
Atorvastatin	Cholesterol synthesis inhibitor	United States	Pre-menopausal women with a strong family history of breast and/or ovarian cancer	Phase II	C	100	NCT00914017	[239]
Vitamin D3	Smoothened inhibitor	Canada	Primary BC	Phase II	C	83	NCT01948128	No results posted
Vitamin D3 + neoadjuvant Progesterone	Apoptotic agent + antiproliferative, cytotoxic	India	Large Operable and Locally Advanced BC	Phase III	ANR	800	NCT01608451	No results posted

**BC**, Breast Cancer; **C**, Completed; **T**, Terminated; **W**, Withdrawn; **HER2-**, human epidermal growth factor receptor 2 negative; **R**, recruiting; **TNBC**, triple-negative breast cancer; **DS**, dietary supplement; **iv**, intravenous; **ANR**, active not recruiting.
